# Low early fixation failure rates with variable-angle locking plate fixation of AO/OTA 34 C patella fractures: a retrospective cohort study

**DOI:** 10.1007/s00590-026-04864-1

**Published:** 2026-07-20

**Authors:** Benyamin Dadpey, Kevin Zhu, Claire Bonnyman, Hevatib Mehmood, William T. Kent

**Affiliations:** Department of Orthopaedic Surgery University of California San Diego, San Diego, USA

**Keywords:** Patella plating, Patella augmentation, Variable angle patella plate, Patella fracture

## Abstract

**Purpose:**

To determine the fixation failure rate of the variable-angle (VA) locking patella plate and to evaluate whether supplemental augmentation reduced the risk of fixation failure.

**Methods:**

A retrospective comparative cohort study was performed at a Level I trauma center between January 2021 and June 2025. Adult patients with OTA/AO 34 C patella fractures treated with a VA locking plate with or without augmentation and ≥ 3 months of follow-up were included. Augmentation was defined as additional plates, independent screws, cerclage wire, heavy non absorbable sutures, or suture anchors that were used to supplement fixation and/or reinforce the inferior patellar pole or extensor mechanism. The primary outcome was fixation failure; secondary outcomes included mode of failure, infection and symptomatic implant removal.

**Results:**

Thirty-four patients were included (17 VA-only, 17 VA + augmentation). Groups were comparable for age, sex, Charlson Comorbidity Index, OTA/AO subclassification, open fracture status and follow-up duration (all *p* > 0.05). The overall fixation failure rate was 2.9% (1/34), occurring at the distal fixation interface in the augmented group. There was no significant difference in failure rate between groups (VA-only 0.0% vs. VA + augmentation 5.9%; *p* = 1.0). One patient in the VA group suffered a fracture-related infection and one patient in the VA + augmentation cohort had a suture abscess. One patient in the VA + augmentation cohort required hardware removal for symptomatic hardware. All remaining patients healed without complications.

**Conclusion:**

Variable-angle locking plate fixation of AO/OTA 34 C patella fractures was associated with low rates of early fixation failure and symptomatic hardware removal. With only a single documented fixation failure, the study was underpowered to draw conclusions regarding the protective role of augmentation on fixation failure.

## Introduction

Patella fractures represent up to 1.5% of all fractures and are the second most common knee fracture [1, 2]. Not all patella fractures warrant surgical fixation. Those resulting in disruption of the extensor mechanism, more than 2 to 3 mm of articular step-off, more than 2 to 4 mm fragment displacement, intra-articular comminution or open fractures, are relative indications for surgical fixation [3]. 

Simple transverse patella fractures are typically managed using tension band wiring (TBW) with cannulated screws or Kirschner wires. In contrast, no consensus exists regarding the optimal fixation strategy for comminuted patella fractures [4, 5]. Modified TBW techniques incorporating lag screws and cerclage wires or sutures have been described for the open reduction and internal fixation (ORIF) of comminuted patella fractures but are associated with failure rates as high as 34%, frequently requiring return to the operating room [6, 7]. 

More recently, plating has gained momentum in the treatment of comminuted patella fractures as it allows capture and reduction of small, comminuted fragments that are not amenable to traditional TBW techniques. Various plating options including mesh and mini-fragment plates along with pre-contoured fixed-angle and variable-angle plates have been described in isolation or in conjunction with additional augmentation for comminuted patella fractures [8–13]. 

One such implant is the patella-specific 2.7 mm pre-contoured variable-angle (VA) locking plate system (Synthes, Paoli, PA). This plates design features including radially arranged arms, distally extending legs, and variable-angle locking screws. The VA plate can be bent and trimmed intraoperatively, on the back table and in situ. This allows it to be tailored to patient-specific fracture geometry and improve its ability to capture of small comminuted and inferior pole fragments [11]. Despite these design advantages, there are limited and conflicting reports in the literature regarding the VA plate’s fixation failure rate when used to treat comminuted patella fractures and whether additional augmentation is necessary to mitigate fixation failure [12, 13]. The purposes of this study were to (1) determine the fixation failure rate of VA patellar plates and (2) evaluate whether augmentation provides additional protection against fixation failure. It was hypothesized that VA plating alone would result in a low fixation failure rate and that supplemental augmentation would not significantly reduce failure compared with isolated VA plating.

## Methods

Upon obtaining institutional review board approval, a retrospective review of all patients who underwent operative fixation of patella fractures at a Level I trauma center between January 1st, 2021, and June 30th, 2025, was conducted. Patella fractures were classified using the Arbeitsgemeinschaft für Osteosynthesefragen (AO) / Orthopaedic Trauma Association (OTA) classification system. Patients with AO/OTA 34 C patella fractures that underwent ORIF patella-specific 2.7-mm pre-contoured variable-angle (VA) locking plate system (Synthes, Paoli, PA) within two weeks of injury were included in the study. Patients that were: (1) younger than 18 years of age, (2) treated non-operatively, (3) were treated with a non-VA plate implant, or (4) followed up less than 3 months were excluded.

Patients who met inclusion criteria were then divided into two groups: (1) treated with VA plate alone or (2) treated with VA plate and additional augmentation. Augmentation was defined as any additional plates, screws, wires, heavy non absorbable suture (cerclage or Krakow type suture into the patellar tendon), or suture anchors used in conjunction with the VA plate to supplement fracture fixation and/or reinforce the inferior pole or extensor mechanism. Operative notes, implant logs, and radiographic images were reviewed to identify the supplemental fixation. Types of augmentation to fixation included: independent interfragmentary screw fixation, mini-fragment plates, circumferential cerclage wires, heavy non absorbable sutures used for patellar tendon fixation, transosseous sutures, and suture anchors.

Electronic medical records and radiologic images were reviewed to obtain patient demographics including past medical history, social history, fracture pattern, postoperative protocols including weight-bearing and range of motion restrictions, implant failure, and mode of failure. Fracture morphology was characterized beyond the AO/OTA classification and included inferior (distal) pole comminution, the number of articular fragments, patellar tendon insertion involvement, and medial and lateral retinacular injury. Inferior pole comminution and articular fragment number were assessed on preoperative CT when available and on radiographs otherwise; retinacular injury was documented from the operative report. Postoperatively, all patients were placed in a hinged knee brace. The brace was maintained in full extension for approximately the first 2 weeks, after which range of motion was advanced approximately by 30° every 2 weeks until full motion was achieved. This protocol was applied uniformly to both treatment groups. Infection was classified using the Fracture-Related Infection (FRI) Consensus Definition; where retrospective data did not permit full FRI classification, infections were described according to the available clinical, laboratory, and microbiological findings [14]. The primary outcome was fixation failure, defined as loss of fixation with a non-intact extensor mechanism. Secondary outcome was mode of implant failure and other postoperative complications including need for removal of symptomatic implants, and superficial and deep infections.

Statistical analyses were performed using Microsoft Excel (Microsoft Corp., Redmond, WA, USA). Continuous variables were assessed for normality using the Shapiro–Wilk test. Given the small sample size and the non-normal distribution of continuous variables, data were presented as median and interquartile range (IQR) and between-group comparisons were performed using the Mann–Whitney U test. Categorical variables were summarized as absolute counts and percentages and were compared between cohorts using Fisher’s exact test. Augmentation subtype data within the augmented cohort were summarized descriptively as counts and percentages. All tests were two-sided, and statistical significance was set at *P* < 0.05.

## Results

### Patient and fracture characteristics

A total of 45 patients were identified who underwent ORIF of patella fractures with VA locking plates. Thirty-four patients had at least 3 months of follow-up and were included in this study. Seventeen patients underwent fixation with the VA plate alone, whereas 17 underwent fixation with the VA plate plus additional augmentation. Patient demographics, and fracture morphology in the VA and VA + augmentation cohorts are presented in Table 1. The VA and VA + augmentation cohorts were comparable in age (median 62 years [IQR, 43–66] vs. 46 years [IQR, 33–71], *p* = 0.53) and sex distribution (64.7% female vs. 52.9% female, *p* = 0.73), BMI (median 27.6 kg/m² [IQR, 25.8–30.1] vs. 24.9 kg/m^2^ [IQR, 24.3–29.5], *p* = 0.11), Charlson Comorbidity Index (CCI) (median 2 [IQR, 0–3] vs. 0 [IQR, 0–3], *p* = 0.59), American Society of Anesthesiologists physical status classification system (ASA), as well as rates of osteoporosis, smoking, drug use, and alcohol use. Both cohorts were also comparable in the rate of open fractures (11.8% vs. 5.9%, *p* = 1.00), and AO/OTA fracture classification (34C1: 5.9% vs. 5.9%; 34C2: 41.2% vs. 23.5%; 34C3: 52.9% vs. 70.6%; *p* = 0.73). Distal pole comminution was present in 17 of 34 fractures (50.0%). Although not statistically significant, there was a trend toward more frequent distal pole comminution in the augmentation group (11/17, 64.7%) compared with the VA-only group (6/17, 35.3%; *p* = 0.17). The median number of articular fragments was 4 (IQR, 3–6) and likewise did not differ significantly between groups (VA-only 4 [IQR, 3–5] vs. augmentation 5 [IQR, 3–6]; *p* = 0.19). Medial and lateral retinacular tears were documented intraoperatively in 26 (76.5%) and 27 (79.4%) patients, respectively, with no significant difference between groups (medial: VA-only 14/17 vs. augmentation 12/17, *p* = 0.69; lateral: VA-only 15/17 vs. augmentation 12/17, *p* = 0.40).

**Table 1:** Patient demographics and patella fracture characteristics

	VA plate (*n* = 17)	VA plate + augmentation (*n* = 17)	Total (*n* = 34)	*P*-value
Age	62 (43–66)	46 (33–71)	59 (37–68)	0.53
Sex, female	11 (64.7)	9 (52.9)	20 (58.8)	0.73
BMI (kg/m²)	27.6 (25.8–30.1)	24.9 (24.3–29.5)	27.13 (24.5–29.7)	0.11
CCI	2 (0–3)	0 (0–3)	2 (0–3)	0.59
ASA 1	1 (5.9)	6 (35.3)	7 (20.6)	0.13
ASA 2	10 (58.8)	8 (47.1)	18 (52.9)	
ASA 3	6 (35.3)	3 (17.6)	9 (26.5)	
Osteoporosis	0 (0)	3 (17.6)	3 (8.8)	0.23
Active smoking	2 (11.8)	3 (17.6)	5 (14.7)	1.00
Recreational drug use	2 (11.8)	0 (0)	2 (5.9)	0.49
Alcohol use	3 (17.6)	1 (5.9)	4 (11.8)	0.60
Open fracture status	2 (11.8)	1 (5.9)	3 (8.8)	1.00
AO/OTA, 34C1	1 (5.9)	1 (5.9)	2 (5.9)	0.73
AO/OTA, 34C2	7 (41.2)	4 (23.5)	11 (32.4)	
AO/OTA, 34C3	9 (52.9)	12 (70.6)	21 (61.8)	
Inferior pole comminution	6 (35.3)	11 (64.7)	17 (50.0)	0.17
Articular fragments	4 (3–5)	5 (3–6)	4 (3–6)	0.19
Medial retinacular repair	14 (82.4)	12 (70.6)	26 (76.5)	0.69
Lateral retinacular	15 (88.2)	12 (70.6)	27 (79.4)	0.40

^a^ Abbreviation: IQR = Interquartile range; BMI = Body mass index; CCI = Charlson comorbidity Index; ASA = American Society of Anesthesiologists physical status classification system; AO/OTA = Arbeitsgemeinschaft für Osteosynthesefragen (AO) / Orthopaedic Trauma Association (OTA).

^b^ Values are presented as n (%) for categorical variables and median (Interquartile range) for continuous variables.

### Operative treatment

ORIF of patellar fractures was performed by four trauma fellowship trained orthopedic surgeons at a single Level I academic trauma center. The VA locking plate was applied to the anterior surface of the patella. Both the three-hole and six-hole leg configurations were used, selected according to the extent of inferior pole comminution, and the plate was contoured in all patients to match the fracture geometry. In addition to anterior-to-posterior locking screws used to capture fragments and secure the plate, in all patients, Inferior-to-superior or superior-to-inferior screws were passed through the leg holes to resist the quadriceps or patellar tendon deforming forces of the patella.Among the 34 patients in whom VA plate was used for ORIF of patella fracture, 50% required augmentation with the VA plate. The decision to augment fixation was made intraoperatively at the discretion of the treating surgeon, based on fracture morphology after VA plate fixation. Augmentation comprised two categories: reductive (bony) augmentation which encompassed independent interfragmentary screws, mini-fragment plates, or cerclage wire that was used to reduce and stabilize comminuted fragments not adequately captured by the plate; and extensor-mechanism augmentations including Krackow patellar tendon sutures or transosseous suture anchors/tunnels which were used to reinforce the patellar tendon insertion in comminuted inferior-pole fractures (Table 2).

**Table 2:** Augmentations used with the VA plate in the VA plate + augmentation cohort

Augmentation type	*n* (%)
Independent screws	11 (64.7)
Additional mini-fragment plate	1 (5.9)
Cerclage wire	1 (5.9)
Patellar tendon krackow suture	10 (58.8)
Trans osseous sutures anchors / Tunnels	2 (11.8)

### Postoperative outcomes

Postoperatively, all patients were immobilized in a brace locked in extension. The knee extension ROM progression is summarized in Table 3.

**Table 3:** Post-operative knee range of motion restriction progression

Knee range of motion restriction	Weeks postoperatively
Brace restricted to 0–30°	2.4 (2.1–2.9)
Brace restricted to 0–60°	4.7 (4.1–5.0)
Brace restricted to 0–90°	6.9 (6.5–7.7)
Brace unlocked	8.6 (7.7–9.5)
Brace discontinued	12.9 (9.8–14.1)

^a^ Values are presented as median (Interquartile range).

Zero patients (0%) in the VA group and three patients in the VA + augmentation group were made non-weight-bearing (NWB) immediately postoperatively owing to concomitant injuries with an ipsilateral femur fracture in two patients and a contralateral pelvic fracture in the other patient. The remaining patients (31/34) were weight bearing as tolerated (WBAT) with the knee fully extended immediately postoperatively. The median overall follow-up duration was 5.37 months (IQR, 4.2–11.7), and it was comparable between VA and VA +augmentation cohorts, 4.47 months (IQR, 4.0–13.4) and 6.50 months (IQR, 4.7–8.6), respectively (*p* = 0.41). At final follow-up, the median knee flexion was 120° (IQR, 100–130°) and was not statistically different between the VA-only (median 115°; IQR, 100–130°) and VA + augmentation cohorts (median 120°; IQR, 110–130°; *p* = 0.54). An active extensor lag was absent in 31 of 34 patients (91.2%); two patients in the VA-only cohort had a 5° lag, and one patient in the VA + augmentation cohort had a 10° lag. All 34 patients were able to perform a straight leg raise against gravity at the last follow up. Of note, the patient with the 10° active extension lag did not adhere to the prescribed range-of-motion protocol, kept the brace locked in full extension, and reached only 25° of flexion at 4.5 months postoperatively. Overall fixation failure rate was 2.9% (1/34), including 0% in the VA plate alone group and 5.9% in the VA + augmentation group. (Table 4). Nine patients (26.5%) had at least 12 months of follow-up and five (14.7%) at least 24 months (maximum, 49 months); no late fixation failures occurred among these patients.

**Table 4:** Post-operative weight bearing restrictions and fixation failure rate

	VA plate	VA plate + augmentation	Total	*p*-value
WBAT in extension	17 (100)	14 (82.4)	31 (91.2)	0.23
NWB in extension	0 (0)	3 (17.6)	3 (8.8)	
Follow-up, (months)	4.47 (4.0–13.4)	6.50 (4.7–8.6)	5.37 (4.2–11.7)	0.41
Fixation failure	0 (0)	1 (5.9)	1 (2.9)	1.00

^a^ Abbreviations: WBAT = Weight bearing as tolerated; NWB = non-weight bearing; IQR = Interquartile range.

^b^ Values are presented as n (%) for categorical variables and median (Interquartile range) for continuous variables.

**Fig. 1:**
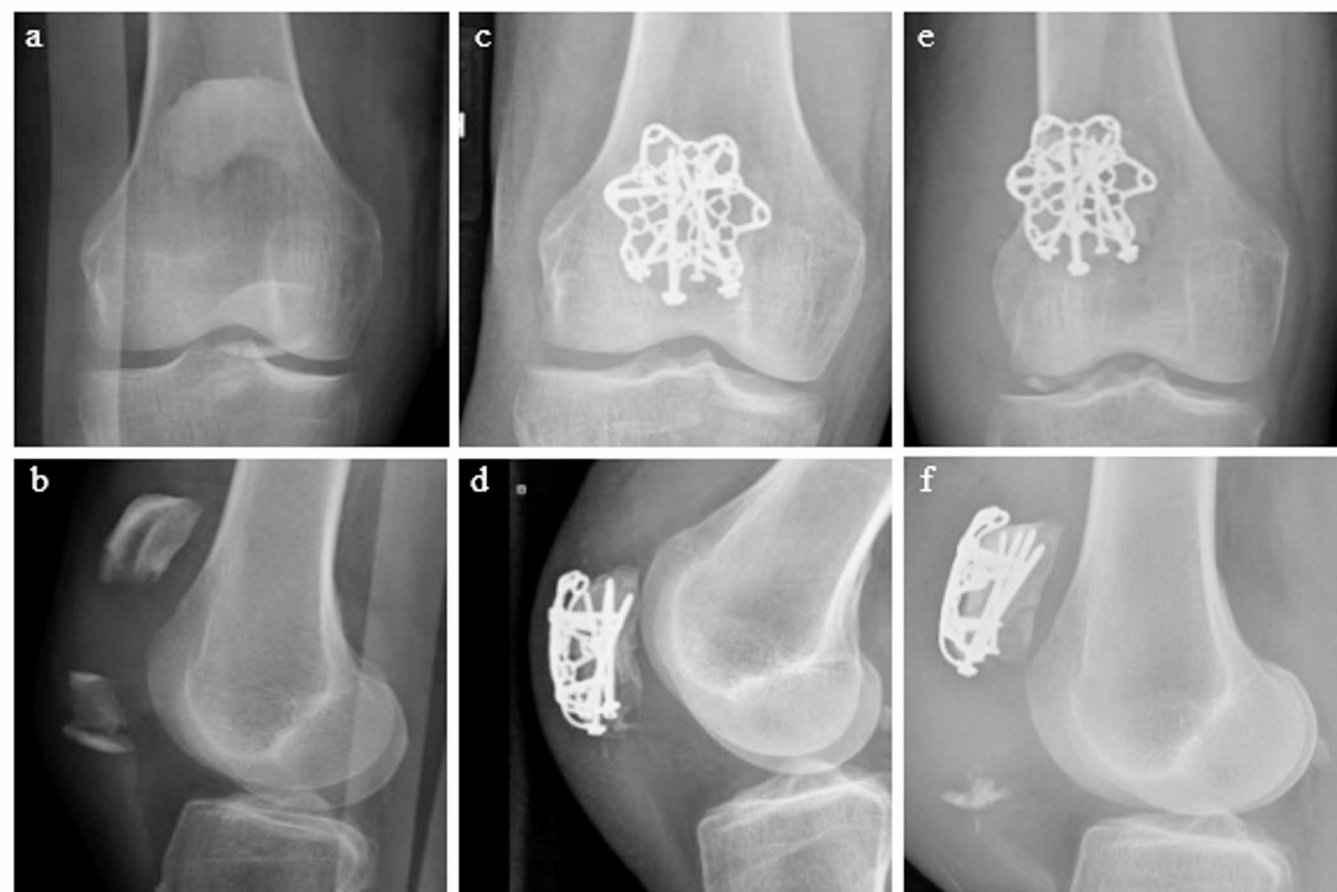
Anterior-posterior and lateral Injury (a & b), post operation (c & d) and fixation failure (e & f) radiographs of the patient who suffered distal pole fixation failure

The single fixation failure occurred in a patient with a comminuted AO/OTA 34C3 fracture involving the inferior pole, treated with a variable-angle locking plate with inferiorly positioned plate legs and axial inferior-to-superior polar screws, supplemented by an additional interfragmentary screw; several small distal fragments were incorporated into the repair construct using sutures. No dedicated patellar tendon (Krackow) reinforcement was performed. Postoperatively the patient was weight-bearing as tolerated in a brace locked in extension per the standard protocol but self-discontinued the brace at approximately 7 weeks. At approximately 8 weeks postoperatively, the construct failed with avulsion of the distal pole and patellar tendon, resulting in patella alta and extensor mechanism disruption (Fig. 1). Revision was performed with removal of the inferior-to-superior polar screws and a Krackow patellar tendon repair using transosseous sutures. At the most recent follow-up, 2 months after revision, the fracture was continuing to heal, with a range of motion of 0–100° and no extensor lag. The Fixation failure rate for AO/OTA 34C3 fracture patterns was 4.8%. One patient in the VA plate group had a deep infection which required operative debridement and subsequently healed uneventfully. One patient in the VA-only group developed a fracture-related infection, meeting FRI confirmatory criteria based on purulent drainage at surgery and two concordant deep-tissue cultures. This patient was managed with operative irrigation and debridement. One patient in the VA + augmentation group developed a suture abscess that did not meet FRI confirmatory criteria and resolved with local wound care. Finally, one patient in the VA + augmentation cohort had symptomatic implants and underwent hardware removal after complete union.

## Discussion

Treatment of patella fractures remains a challenge to orthopedic surgeon and to date, no consensus exists for the optimal fixation construct in the treatment of comminuted patella fractures [12, 13, 15–20]. This study reported the outcomes of a variable-angle (VA) plate with and without supplemental augmentation in the treatment of AO/OTA 34 C patella fractures.

The overall fixation failure rate of the VA plate in AO/OTA 34C1, 34C2 and 34C3 patella fractures was low (2.9%). No significant difference in fixation failure was detected between fixation with and without augmentation (0% vs. 5.9%; *p* = 1.00); however, given the single failure event and the resulting lack of statistical power, this absence of a detected difference should not be interpreted as evidence of equivalence, and no conclusion can be drawn regarding whether augmentation reduces fixation failure. In the more comminuted C3 fractures, fixation failure was also low (4.8%). A fracture-related infection occurred in one patient (VA-only group) and a suture abscess not meeting FRI criteria in one patient (VA + augmentation group); symptomatic hardware requiring removal occurred in one patient (VA + augmentation group). These complications were comparable between the groups.

Fixation failure continues to be a concern following fixation of patella fractures [12, 15, 17]. Comminuted patella fractures are challenging to fix using traditional tension band (TB) techniques such as wiring or cannulated screws with suture or cables, as the tension band construct requires an intact or reconstructable compression side. With comminution, TB wires or screws alone are often insufficient to secure and stabilize all comminuted fragments [19, 21]. To address this challenge, mesh plating was introduced as it allowed for multiplanar fracture fixation and stabilization of the distal inferior pole comminution that is seen in 88% of comminuted patella fractures [22]. Lorich et al. demonstrated the use of mesh plating in treating 9 cases of AO/OTA 34C3 comminuted patella fractures resulting in no fixation failure and a 11% symptomatic hardware removal rate [17]. Volgas and Dreger reported in a larger series of 16 patients (9 primary ORIF, 7 revision ORIF) treated with mesh plates reported no fixation failure but a high 32% symptomatic hardware removal rate [18]. Despite its demonstrated effectiveness in treating comminuted patella fractures and low fixation failure rate downsides to mesh plating are increased operative time required for contouring and cutting the mesh plate intraoperatively and relatively high symptomatic hardware removal rates. To address these issues, pre-contoured locking plates were designed for the treatment of patella fractures [6, 8, 23]. 

Buschbeck et al. reported on the use of a recontoured locking plate (patella SuturePlate™, Arthrex^®^, Naples, USA) in 29 patients for treatment of comminuted AO/OTA 34C3 patella fractures. They reported no failures of fixation and a 24% symptomatic hardware removal rate [20]. Ellwein et al. used the same plate in prospective cohort of 19 patients with patella fractures (45% with comminution) and had a 5% fixation failure rate with a 15% symptomatic hardware removal rate [24]. Wild et al. described the use of another pre-contoured fixed angle patella plate (Koenigsee Implantate, Allendorf, Germany) and reported a 5% fixation failure rate and 5% hardware removal rate [23]. 

The patella-specific 2.7 mm variable angle locking plate evaluated in this study shares several design features with fixed-angle plates, including the ability to place multiple anterior-to-posterior locking screws. However, its 15-degree angular freedom with screw placement allows for more potential fixation points in comminuted fragments. Additionally, the plate legs permit placement of axial superior-to-inferior and inferior-to-superior screws longitudinally along the long axis the patella, facilitating fixation of inferior pole comminution and resistance against the deforming forces of the knee extensor mechanism. Although the plate is anatomically pre-contoured by the manufacturer, it can be further contoured, and cut intraoperatively to accommodate individual fracture morphology. These design features position the VA plate as a theoretically advantageous implant for achieving reliable fracture reduction with low failure rates. 

Two prior studies have examined this specific implant with conflicting results. Shaath et al. used the patella-specific 2.7 mm VA locking plate to treat 61 patella fractures (52% with comminution) and reported an overall fixation failure rate of 3% and a 3.1% failure rate specifically among comminuted AO/OTA 34C3 patella fractures, with no cases of symptomatic hardware requiring reoperation [13]. In contrast, Hoskins et al. used the same implant in 38 AO/OTA 34C3 fractures and reported an overall fixation failure rate of 26%, rising to 40% in patients treated with the VA plate without augmentation, along with a 13% rate of symptomatic hardware removal [12]. 

Our findings of 2.9% fixation failure rate and 2.9% symptomatic hardware removal rate were more consistent with those of Shaath et al. Several shared methodological features may explain these findings. In both studies, all ORIFs were performed by fellowship-trained orthopedic trauma surgeons, axial inferior-to-superior and superior-to-inferior screws were passed through the plate legs in all patients, and postoperative all patients were immobilized in full knee extension [13]. The markedly higher failure rate reported by Hoskins et al. may be attributable to several factors: surgery was performed by 10 different surgeons without trauma fellowship training; postoperative range-of-motion restriction was not uniformly applied; and axial screws through the plate legs were not consistently utilized. Notably, of the ten patients who experienced mechanical failure, nine had unrestricted or only partially restricted initial postoperative range of motion. Moreover, among the nine patient that had no range of motion restrictions postoperatively six patients had fixation failure [12]. This pattern underscores the potential importance of postoperative immobilization in full extension with a limited range of motion arc as a modifiable factor. Although Hoskins et al. attributed their high failure rate primarily to fracture comminution, both our study and that of Shaath et al. demonstrate low failure rates in AO/OTA 34C3 fractures (4.9% and 3.1%, respectively) [12, 13]. 

This study has several limitations inherent to its retrospective design and small sample size. With only 34 patients and a single failure event, the study is underpowered to detect meaningful differences in failure rates between groups. The median follow-up of 5.37 months may not capture late complications; however, no late fixation failures were observed among the nine patients followed beyond 12 months (five beyond 24 months), and most fixation failures following patella ORIF are known to occur early in the postoperative course [12]. Although knee range of motion was recorded, validated patient-reported outcome measures were not collected, which limits characterization of patient-perceived function. Finally, preoperative CT was available in 20 of 34 patients; in the remainder, comminution and articular fragment number were graded on radiographs, which may underestimate the true degree of comminution.

Interpretation of the augmentation comparison is subject to additional limitations. Augmentation was not a single intervention but encompassed two functionally distinct categories: reductive (bony) augmentation aimed at reducing and stabilizing comminuted fragments (independent interfragmentary screws, mini-fragment plates, and cerclage wire), and extensor-mechanism augmentation aimed at reinforcing the patellar tendon insertion in comminuted inferior-pole fractures (Krackow patellar tendon sutures and transosseous suture anchors or tunnels), frequently used in combination. Because these categories address different failure mechanisms, “augmentation” in this cohort does not represent a uniform or reproducible intervention, and our data cannot support generalizable conclusions about augmentation as a category. Moreover, the use and type of augmentation were not randomized but were determined intraoperatively by the treating surgeon on the basis of fracture morphology and construct stability. Consistent with this, extensor-mechanism augmentation was applied exclusively within the augmentation group (8/17 vs. 0/17; *p* = 0.003), and augmentation overall was applied preferentially to more complex injuries, including those with inferior pole comminution (64.7% vs. 35.3%) and AO/OTA 34C3 patterns (70.6% vs. 52.9%). This confounding by indication may obscure a true protective effect of augmentation: because more complex fractures preferentially received augmentation, comparable failure rates may understate any benefit, while the failure rate in the VA-only group may be lower than expected had it included equally complex injuries. We have therefore avoided causal interpretation of augmentation’s effect on fixation failure. A prospective study with standardized augmentation protocols and functional outcomes would help to better define the indications for specific augmentation strategies across fracture morphologies.

## Conclusion

In this small retrospective cohort with short-term follow-up, variable-angle locking plate fixation of comminuted AO/OTA 34 C patella fractures was associated with low rates of early fixation failure and symptomatic hardware removal. Because only a single failure occurred, the study was underpowered to detect a difference between fixation with and without augmentation, and no conclusion regarding the value of augmentation can be drawn.

## Data Availability

No datasets were generated or analysed during the current study.
